# KatE From the Bacterial Plant Pathogen *Ralstonia solanacearum* Is a Monofunctional Catalase Controlled by HrpG That Plays a Major Role in Bacterial Survival to Hydrogen Peroxide

**DOI:** 10.3389/fpls.2020.01156

**Published:** 2020-07-31

**Authors:** María Laura Tondo, Roger de Pedro-Jové, Agustina Vandecaveye, Laura Piskulic, Elena G. Orellano, Marc Valls

**Affiliations:** ^1^Área Biología Molecular, Facultad de Ciencias Bioquímicas y Farmacéuticas, Universidad Nacional de Rosario, Rosario, Argentina; ^2^Instituto de Ingeniería Ambiental, Química y Biotecnología Aplicada (INGEBIO), Facultad de Química e Ingeniería del Rosario, Pontificia Universidad Católica Argentina (UCA), Consejo Nacional de Investigaciones Científicas y Técnicas (CONICET), Rosario, Argentina; ^3^Centre for Research in Agricultural Genomics (CSIC-IRTA-UAB-UB), Catalonia, Spain; ^4^Department of Genetics, University of Barcelona, Barcelona, Spain; ^5^Instituto de Biología Molecular y Celular de Rosario, Consejo Nacional de Investigaciones Científicas y Técnicas (CONICET), Rosario, Argentina; ^6^Área Estadística y Procesamiento de Datos, Facultad de Ciencias Bioquímicas y Farmacéuticas, Universidad Nacional de Rosario, Rosario, Argentina

**Keywords:** *Ralstonia solanacearum*, bacterial wilt, oxidative burst, KatE catalase, host adaptation

## Abstract

*Ralstonia solanacearum* is the causative agent of bacterial wilt disease on a wide range of plant species. Besides the numerous bacterial activities required for host invasion, those involved in the adaptation to the plant environment are key for the success of infection. *R. solanacearum* ability to cope with the oxidative burst produced by the plant is likely one of the activities required to grow parasitically. Among the multiple reactive oxygen species (ROS)-scavenging enzymes predicted in the *R. solanacearum* GMI1000 genome, a single monofunctional catalase (KatE) and two KatG bifunctional catalases were identified. In this work, we show that these catalase activities are active in bacterial protein extracts and demonstrate by gene disruption and mutant complementation that the monofunctional catalase activity is encoded by *katE*. Different strategies were used to evaluate the role of KatE in bacterial physiology and during the infection process that causes bacterial wilt. We show that the activity of the enzyme is maximal during exponential growth *in vitro* and this growth-phase regulation occurs at the transcriptional level. Our studies also demonstrate that *katE* expression is transcriptionally activated by HrpG, a central regulator of *R. solanacearum* induced upon contact with the plant cells. In addition, we reveal that even though both KatE and KatG catalase activities are induced upon hydrogen peroxide treatment, KatE has a major effect on bacterial survival under oxidative stress conditions and especially in the adaptive response of *R. solanacearum* to this oxidant. The *katE* mutant strain also exhibited differences in the structural characteristics of the biofilms developed on an abiotic surface in comparison to wild-type cells, but not in the overall amount of biofilm production. The role of catalase KatE during the interaction with its host plant tomato is also studied, revealing that disruption of this gene has no effect on *R. solanacearum* virulence or bacterial growth in leave tissues, which suggests a minor role for this catalase in bacterial fitness *in planta*. Our work provides the first characterization of the *R. solanacearum* catalases and identifies KatE as a *bona fide* monofunctional catalase with an important role in bacterial protection against oxidative stress.

## Introduction

*Ralstonia solanacearum* is a gram-negative, soil-borne β-proteobacterium that causes the bacterial wilt disease in more than 200 plant species, including economically important food crops such as potato, tomato, peanut, and eggplant (Allen et al., 2004). In addition to its extremely wide host range, *R. solanacearum* exhibits an increasingly broad geographic distribution and is able to survive for long periods in waterways, soil and in symptomless or latently infected plants (Denny, 2006; Genin and Denny, 2012).

Upon interaction with a susceptible host, the pathogen initiates the infection by entering the roots. After colonisation of the intercellular spaces of the root cortex, the bacterium enters the xylem vessels, spreading rapidly, and systemically through the vascular system. Intensive bacterial multiplication and production of large amounts of exopolysaccharides (EPSs) blocks water traffic in vascular bundles, ultimately resulting in complete wilting, plant death, and the release of the pathogen back to the soil (Genin and Denny, 2012). *R. solanacearum* requires multiple virulence factors that act additively to facilitate infection of the host plant. Bacterial motility mediated by flagella and type IV pili, plant cell wall-degrading enzymes, and type II-secreted proteins enable bacterial penetration into root tissues. Secretion of type III effectors inside plant cells evades plant immune responses and allows disease development (Peeters et al., 2013). In the plant environment, *R. solanacearum* must overcome different types of metabolic stresses in order to survive and proliferate. One of these challenges is the exposure to plant-generated reactive oxygen species (ROS) that accumulate in the apoplast as part of the primary defence response to pathogen invasion (Lamb and Dixon, 1997).

ROS are unavoidable by-products of plant metabolic pathways generated as a result of successive one-electron reductions of molecular oxygen (O_2_). Under physiological steady state conditions, ROS accumulation is prevented by the action of protective antioxidant systems often confined to specific compartments. However, adverse environmental factors including pathogen infection disturb this fine balance between production and scavenging of ROS leading to a rapid increase in intracellular ROS levels or “oxidative burst” (Apel and Hirt, 2004). In plants challenged with pathogenic microorganisms, including fungi, bacteria, and viruses, the oxidative burst proved to be one of the earliest events after elicitation (Wojtaszek, 1997). In the interaction of *R. solanacearum* with tomato plants, a single-phase ROS increase was detected at 24 h post-inoculation (hpi) of a susceptible cultivar, while a bi-phasic ROS generation with peak levels at 12 and 36 hpi was observed after infection of a resistant tomato variety (Mandal et al., 2011). The second phase of ROS accumulation, usually more prolonged and higher in magnitude, has been correlated with disease resistance *via* the hypersensitive response during incompatible and non-host interactions (Lamb and Dixon, 1997).

The oxidative burst fulfils multiple functions to plant cells undergoing pathogen attack. ROS promote the oxidative cross-linking of plant cell walls to slow pathogen entry and spread, and act as key signal molecules that mediate the activation of plant defence responses and systemic resistance (Lamb and Dixon, 1997). In addition, the high reactivity of ROS with cellular macromolecules, including DNA and proteins, make ROS effective antimicrobial agents capable of either killing the pathogen or slowing down its growth (Peng and Kuc, 1992). To counter-attack ROS, oxidative stress response genes were shown to be expressed in plant-associated bacteria during the interaction with their hosts (Smith et al., 1996; Santos et al., 2001; Okinaka et al., 2002; Saenkham et al., 2007; Tamir-Ariel et al., 2007). Particularly, an *in vivo* expression technology (IVET) screen performed in *R. solanacearum* during pathogenesis of tomato plants revealed that at least 15 out of 153 *in planta*-expressed genes encoded proteins involved in the oxidative stress response, further supporting the notion that an oxidative challenge is associated with plant infection (Brown and Allen, 2004; Flores-Cruz and Allen, 2009).

Hydrogen peroxide (H_2_O_2_), the major ROS of the oxidative burst, is an electrically neutral and relatively stable species that can penetrate through cell membranes and diffuse to reach distant cellular components (Wojtaszec, 1997). H_2_O_2_ concentrations must be kept at low levels inside bacterial cells due to its ability to oxidize ferrous ions to generate highly reactive hydroxyl radicals (·OH; Fenton reaction), and to react with iron-sulphur clusters of key metabolic enzymes (Mishra and Imlay, 2012). Among the bacterial enzymes evolved to remove ROS and avoid toxicity, catalases (E.C. 1.11.1.6; H_2_O_2_:H_2_O_2_ oxidoreductase) constitute the primary scavengers of H_2_O_2_ by catalyzing its dismutation to water and oxygen. Based on phylogenetic analyses, three distinct catalase families can be distinguished: typical (monofunctional) heme catalases (KatEs), bifunctional heme catalase-peroxidases (KatGs), and (non-heme) manganese catalases (MnCats) (Zámocky et al., 2012). Most sequenced bacterial genomes encode multiple catalase isozymes that operate in different physiological or environmental conditions (Mishra and Imlay, 2012). Induction of specific catalases has been observed when bacteria detect environmental ROS and upon entry into the stationary phase (Loewen, 1997; Mishra and Imlay, 2012). In addition, recent reports have demonstrated the role of particular catalases during pathogenesis, enhancing the bacterial ability to overcome host-induced oxidative burst (Jittawuttipoka et al., 2009; Tondo et al., 2010; Mishra and Imlay, 2012). The available *R. solanacearum* GMI1000 genome encodes numerous predicted ROS-scavenging enzymes, including three putative catalases. The *RSc0775* (KatGb) and *RSc0776* (KatGa) open reading frames (ORFs) encode predicted bifunctional catalase-peroxidases in the bacterial chromosome; whereas *RSp1581* (KatE) codes for a predicted typical monofunctional catalase and is located in the megaplasmid, which harbors most *R. solanacearum* pathogenicity functions (Salanoubat et al., 2002; Genin and Denny, 2012).

Our previous transcriptomic studies in *R. solanacearum* extracted from roots of early infected potato plants indicated that the transcription of *katE* and, to a lesser extent, *katGb* is induced during plant colonisation compared to growth in rich medium (Puigvert et al., 2017). We also identified *katE* among the genes specifically induced by HrpG, a key *R. solanacearum* pathogenicity regulator that responds to direct bacterial contact with plant cells (Valls et al., 2006). In addition, *R. solanacearum* catalases are up-regulated by the transcriptional regulator OxyR, whose deletion impaired bacterial virulence (Flores-Cruz and Allen, 2011). These observations collectively suggest a role for catalases during the infection process, but the contribution of these enzymes to bacterial wilt disease has not been investigated.

Here we present a thorough study of the *R. solanacearum* KatE. We prove that this gene encodes a *bona fide* catalase enzyme responsible for one of the two catalase activities detected in this pathogen, describe its expression pattern and study its role during bacterial life *in planta*.

## Materials and Methods

### Bacterial Strains, Plasmids, and Growth Conditions

Relevant characteristics of the plasmids and bacterial strains used in this work are described in **Table 1**. The wild-type strain GMI1000 of *R. solanacearum* and its *hrpG*-derivative have been previously described (Boucher et al., 1985; Valls et al., 2006). The complemented (*ΔhrpG* + *hrpG*) strain was obtained by electroporation of the *ΔhrpG* mutant with pLT-HrpG, a vector that overexpress HrpG from the isopropyl-b-D-thiogalactopyranoside (IPTG)-inducible *Ptac* promoter. The plasmid and transformation procedures are described in (Valls et al., 2006). *R. solanacearum* strains were routinely grown at 28°C in tetrazolium chloride (TZC) agar plates (Kelman, 1954), complete BG medium (10 g/L bactopeptone, 1 g/L yeast extract, 1 g/L casamino acids, 0.5% glucose), or MP minimal medium supplemented with 20 mM L-glutamate as a carbon source (Plener et al., 2010). To induce HrpG expression in the complemented *ΔhrpG* + *hrpG* strain IPTG was added to the cultures at a final concentration of 100 mM. Gentamicin and tetracycline were used for selection of *R. solanacearum* strains (5 and 10 µg/mL in liquid and solid cultures, respectively). Bacterial growth was monitored by measuring optical density at 600 nm. *Escherichia coli* strains were grown at 37°C in Luria-Bertani (LB) broth supplemented with appropriate antibiotics (Sambrook and Russell, 2001).

**Table 1:** Bacterial strains, plasmids, and primers used in this study.

Strain/plasmid	Relevant genotype and description	Source/reference
***Ralstonia solanacearum***		
GMI1000	Wild-type strain	Boucher et al., 1985
*ΔkatE*	*katE* deletion mutant in the GMI1000 background, Gm^r^	This study
*ΔkatE + katE*	*ΔkatE* strain complemented with *katE* from pRCT-katE, Gm^r^, Tc^r^	This study
*ΔhrpG*	*hrpG* deletion mutant in the GMI1000 background	Valls et al., 2006
*ΔhrpG + hrpG*	*ΔhrpG* strain complemented with the overexpressing plasmid pLT-HrpG, Tc^r^	This study
***Escherichia coli***		
JM109	*HsdR17 endA1 recal thi gyrA96 relA1 recA1 supE44* *λ^-^Δ(lac-proAB)*, [F’, traD36, proA^+^B^+^, *lacI^q^ZΔM15*]	Promega Corp.
**Plasmids**		
pGEM-T Easy	PCR cloning and sequencing vector, Ap^r^	Promega Corp.
pGEM-UkatE	PCR-amplified (945-bp) *katE* upstream fragment, cloned in pGEM-T easy, Ap^r^	This study
pGEM-DkatE	PCR-amplified (892-bp) *katE* downstream fragment, cloned in pGEM-T easy, Ap^r^	This study
pCM351	Allelic exchange vector, Ap^r^, Tc^r^, Gm^r^	Marx and Lidstrom, 2002
pCM-UDkatE	Upstream (945-bp) and downstream (892-bp) fragments of *katE* cloned into *Eco*RI/*Not*I and *Hpa*I/*Sac*I sites of pCM351, Ap^r^, Tc^r^, Gm^r^	This study
pRCT	pRC containing tetracycline resistance and cloning sites, Ap^r^, Cl^r^, Tc^r^	Monteiro et al., 2012b
pRCT-katE	PCR-amplified (1960-bp) fragment containing *katE* ORF and promoter sequence, cloned into *Hpa*I/*Bgl*II sites of pRCT, Ap^r^, Cl^r^, Tc^r^	This study
pLT-HrpG	pLAFR3 derivative including the *HrpG* coding sequence under the control of the *Ptac* promoter	Valls et al., 2006
pJBA128	Vector containing *gfpmut3* under a constitutive *PlacUV5* promoter, Tc^r^	Lee et al., 2005
**Primer name**	**Sequence^a^**	**Amplified fragment**
katEU-F	5’ tagaattcGGATACTGACCGTTGCCATC 3’ (*Eco*RI)	This study
katEU-R	5’ tagcggccgcGAGTCTCCTGTGGGGATGAG 3’ (*Not*I)	This study
katED-F	5’ tagttaacGCTGCAGGACTGATGATGTG 3’ (*Hpa*I)	This study
katED-R	5’ tagagctcGGTCACGGATATCGAACCAC 3’ (*Sac*I)	This study
UkatEU-F	5’ GAATGCTTTCCGCCTTGATATC 3’	This study
Gent-R	5’ CCTGCTGCGTAACATCGTTGC 3’	This study
ckatE-F	5’ tagttaacTGTTTGAAGACGGTGACGTT 3’ (*Hpa*I)	This study
ckatE-R	5’ taagatctTCAGTCCTGCAGCTTCG 3’ (*Bgl*II)	This study
katE_qPCR-F	5’ TGAACAAGAACCCGGAGAAC 3’	This study
katE_qPCR-R	5’ TGTCGGCATACGAGAAGATG 3’	This study

Ap^r^, Gm^r^, Tc^r^: resistance to ampicillin (Ap), gentamicin (Gm) and tetracycline (Tc), respectively; PCR, polymerase chain reaction.

^a^Capital letters correspond to nucleotides of the R. solanacearum GMI1000 genome sequence and small letters to nucleotides added to facilitate cloning (restriction sites underlined).

### Molecular Biology and Microbiological Techniques

Molecular cloning procedures, including DNA restriction and analysis, DNA ligation, preparation of competent cells, and transformation of *E. coli* by electroporation, were performed according to standard protocols (Ausubel et al., 1994; Sambrook and Russell, 2001). Plasmid DNA was isolated using Wizard Plus SV Minipreps DNA Purification System (Promega Corp., Madison, WI). Restriction enzymes, DNA ligase, and other DNA enzymes were used according to the manufacturers’ recommendations. Total genomic DNA from *R. solanacearum* was isolated from fresh bacterial cultures as described by Chen and Kuo (Chen and Kuo, 1993). For RNA extraction and quantitative real-time PCR analysis, total RNA was extracted using the SV Total RNA Isolation Kit (Promega) following manufacturer’s instructions for Gram-negative bacteria. cDNA was synthesized using the High Capacity cDNA reverse transcriptase kit (Applied Biosystems) following manufacturer’s instructions. The Sybr Green Master Mix (Sigma Aldrich) was used for quantitative real-time PCR with the LightCycler 480 Instrument (Roche Life Science) using the katE_qPCR-F and katE_qPCR-R oligonucleotides as primers. Per each biological condition, duplicates were run and the phosphoserine aminotransferase gene (*serC*) was used as a reference gene for normalisation of expression as described in (Monteiro et al., 2012a).

### Construction of the *R. solanacearum ΔkatE* Mutant and Complemented Strain

The *R. solanacearum ΔkatE* mutant strain was generated by inserting a gentamicin resistance cassette in substitution of the open reading frame *RSp1581* in the GMI1000 strain. Primers were designed in order to amplify 945-bp (primer pair katEU-F/katEU-R) and 892-pb (primer pair katED-F/katED-R) fragments located upstream and downstream of the gene *RSp1581*, respectively (**Table 1**). Specific restriction sites were incorporated to each primer to be used in subsequent cloning steps. PCR amplifications were performed with the proofreading Phusion DNA polymerase (New England Biolabs, Inc., Ipswich, MA, U.S.A.) following the manufacturer’s conditions. The resulting fragments were cloned into pGEM-T easy (Promega Corp.) creating pGEM-UkatE and pGEM-DkatE for the upstream and downstream regions of the *katE* gene, respectively; and the identity of the inserts were confirmed by sequencing with vector primers SP6 and T7. Inserts were then excised by double digestion with *Eco*RI/*Not*I (upstream region) and *Hpa*I/*Sac*I (downstream region), and inserted into the multiple cloning sites of pCM351 (Marx and Lidstrom, 2002) on both sides of the gentamicin resistance cassette, creating pCM-UDkatE. This construction was then linearized by *Eco*RI and introduced into the wild type *R. solanacearum* GMI1000 by natural transformation following the protocol described by Boucher and associates (Boucher et al., 1985). Double recombination events were selected by gentamicin resistance on TZC agar plates and the correct insertion in the genome was confirmed by PCR using primers UkatEU-F and Gent-R, which hybridize upstream of the upper region used for the homologous recombination and in the gentamicin resistance cassette, respectively (**Table 1**). This mutant strain, denoted as *ΔkatE*, was used for phenotypic characterization.

For *ΔkatE* complementation, a 1960-bp DNA fragment containing the *katE* coding region and extending 430 pb upstream of the 5’ end of the ORF was PCR amplified with primers ckatE-F and ckatE-R (**Table 1**). The amplified sequence included the putative promoter region of the *katE* gene as predicted with SoftBerry (www.softberry.com). This amplicon was double digested with *Hpa*I/*Bgl*II and cloned into the integration element of the suicide vector pRCT (Monteiro et al., 2012b) to generate recombinant plasmid pRCT-katE. This plasmid was then linearized by *Nco*I and introduced into the mutant strain *ΔkatE* by natural transformation as described above. Complemented strains were selected by tetracycline resistance on TZC agar plates. The complemented mutant strain selected for further studies was designated *ΔkatE* + *katE*.

### Enzyme Activity Assay and Staining

*R. solanacearum* soluble cell extracts were prepared from 10 mL cultures harvested by centrifugation at 4,000 *g* for 10 min at 4°C. Bacteria were washed and resuspended in 500 μL of ice-cold 50 mM potassium phosphate buffer (pH 7.0) containing 1 mM PMSF, and then disrupted by intermittent sonication. Suspensions were clarified by centrifugation at 12,000 *g* for 20 min at 4°C. Protein concentrations in soluble cell extracts were determined by the Sedmak and Grossberg method (Sedmak and Grossberg, 1977) with bovine serum albumin as standard. Catalase activity in cell extracts was monitored through the decomposition of hydrogen peroxide by following the decrease in absorbance at 240 nm (Beers and Sizer, 1952). The assays were performed at 25°C in 50 mM potassium phosphate buffer (pH 7.0) containing 10 mM H_2_O_2_. To calculate the catalase specific activity an extinction coefficient of 43.6 M^-1^ cm^-1^ at 240 nm was used. One unit of catalase activity was defined as the amount of activity required to decompose 1 μmol of H_2_O_2_ per minute under the assay conditions.

For evaluation of catalase activity in gels, soluble protein extracts (15–25 μg) were separated by continuous electrophoresis in 8% (w/v) non-denaturing polyacrylamide gels in glycine buffer (pH 9.5). To eliminate the likelihood of multiple, potentially artifactual catalase bands, non-denaturing gels were electrophoresed at a constant current of 10 mA. Staining for catalase activity was performed as previously described (Scandalios, 1968).

Peroxidase activity staining was performed according to Kang and associates (Kang et al., 1999) with some modifications. Briefly, aliquots of cell extracts containing 100 μg of soluble protein were electrophoresed on 8% (w/v) native polyacrylamide gels as previously described. Gels were then incubated in 0.1 M Tris-HCl (pH 7.5) containing 0.1 mg/mL 3,3’-diaminobenzidine, 9 mM H_2_O_2_, and 0.4 mg/mL NiCl_2_ for approximately 30 min in the dark, until appearance of the bands.

Coomassie-stained gels were run in parallel to those used for catalase and peroxidase activity measurements to ascertain comparable protein loadings between samples.

### Bacterial Survival in the Presence of Hydrogen Peroxide

To test bacterial resistance to hydrogen peroxide *R. solanacearum* overnight cultures were inoculated into fresh BG medium and grown to early exponential phase (6.5 h at 28°C and 200 rpm). Aliquots of the cultures were diluted and plated on BG-agar in order to quantify the bacterial population and then hydrogen peroxide was added to the cultures at final concentrations of 1 and 2.5 mM. After 15 min of exposure to the oxidant, samples were removed, washed once with fresh medium, serially diluted and plated on BG-agar plates.

For the induction experiments, *R. solanacearum* cultures were grown to early exponential phase (6.5 h) and incubated with sub-lethal concentrations of hydrogen peroxide (25, 50, and 100 μM) for an additional hour before being used in the killing experiments. After the induction treatment, aliquots of the cultures were washed, diluted and plated on BG-agar plates. Cultures were then treated with a lethal concentration of H_2_O_2_ (5 mM) for 15 min, after which samples were taken, washed once with fresh medium, serially diluted and plated on BG-agar plates.

In all cases, growth of liquid cultures was monitored spectrophotometrically by optical density at 600 nm (OD_600_). Colonies were counted after 72 h incubation at 28°C. The percentage of survival was defined as the number of colony forming units (CFU) after treatment divided by the number of CFU prior to treatment ×100.

### Biofilm Observation and Quantification

For analyses of biofilm formation *R. solanacearum* strains were modified to express the green fluorescent protein (GFP) by electroporation with plasmid pJBA128 (Lee et al., 2005). Saturated cultures of the GFP-labeled bacteria in BG medium were adjusted to an optical density at 600 nm of 0.1 and diluted 1:20 in fresh CPG medium (1 g/L casamino acids, 5 g/L glucose and 10 g/L bacteriological peptone). Then, 300 μL of the bacterial suspensions were placed onto chamber-covered glass slides (nu155411, Lab-Tek, NUNC, Naperville. IL, U.S.A.) that were statically incubated in a humidified PVC box at 28°C. All microscopic observations were performed on a Zeiss LSM880 confocal laser scanning microscope (Carl Zeiss, Jena, Germany) equipped with an argon laser and detector and ﬁlter sets for monitoring of GFP expression (excitation, 488 nm; emission, 517 nm). The images obtained were analyzed with ImageJ software (https://imagej.nih.gov).

Biofilm quantification analyses were carried out following crystal violet assay. In short, CPG overnight cultures were adjusted in CPG to an OD_600_ of 0.1. Next, 95 µL of fresh CPG and 5 µL of the adjusted culture to OD_600_ of 0.1 were added in each of the 96-well polystyrene microplates (Greiner, Kremsmünster, Austria) and incubated without shaking at 30°C during 24 h. After incubation, biomass growth was measured at OD_600_. Next, 100 µL of 0.1% crystal violet stain was added to each well and incubated at room temperature for 30 min. Wells were washed three times with MQ-water and the stained biofilm was solubilised with 100 µL of 95% ethanol and measured at OD_580_. Measurements were performed using SpectraMax multi-plate reader and the results normalised to biomass (OD_580_/OD_600_).

### Pathogenicity and Bacterial Multiplication Assays in Tomato

The susceptible tomato (*Solanum lycopersicum*) cv. Marmande cultivar was grown under long-day light conditions at 25°C and 60% relative humidity. Prior to infection, three- to four-week-old plants were acclimated for 3 days at 27°C with constant light conditions (12 h light/12 h darkness). For the pathogenicity assays, plants not watered for two days were drench inoculated without root wounding with 40 mL of the bacterial suspension adjusted to 10^7^ CFU/mL from an overnight culture. 20–25 plants were inoculated per strain and wilting symptoms were recorded per plant using an established semi-quantitative wilting scale ranging from 0 (no wilting) to 4 (death) (Monteiro et al., 2012b).

For bacterial growth assays *in planta*, tomato leaves were vacuum-infiltrated submerging the aerial plant into water or 10^5^ CFU/mL bacterial suspensions for 20 s. In both cases, the adjuvant Silwet L-77 was added (80 μL/L suspension) to facilitate infiltration. At day 0 and 3 post infiltration, bacterial concentrations in the plant tissues were measured. To this end, three 5-mm diameter disks per biological replicate were taken from infiltrated leaves, homogenised and 10 µL of serial ten-fold dilutions were plated in selective plates. The plates were incubated at 28°C until colonies could be counted. Three biological replicas were used per bacterial strain.

### Statistical Analyses

Quantitative analyses were performed with at least three independent biological samples. Data were subjected to a multifactorial mixed model ANOVA and Tukey’s multiple comparison tests along with residual analysis and validation using Infostat software (Infostat 2006H, http://www.infostat.com.ar).

## Results

### *R. solanacearum RSp1581* Encodes an Active Monofunctional Catalase Induced During Exponential Growth

In order to investigate the role of *katE* in the *R. solanacearum* physiology and plant interaction, we generated a *katE* deletion mutant by genetic replacement of the *RSp1581* open reading frame by a gentamicin resistance cassette in the GMI1000 strain background. The resulting mutant, named *ΔkatE*, exhibited typical colony morphology on tetrazolium chloride (TZC)-containing agar plates and similar growth curves to its wild-type parental strain, demonstrating that disruption of *katE* does not affect bacterial growth *in vitro* (**Supplementary Figure 1**).

**Figure 1:**
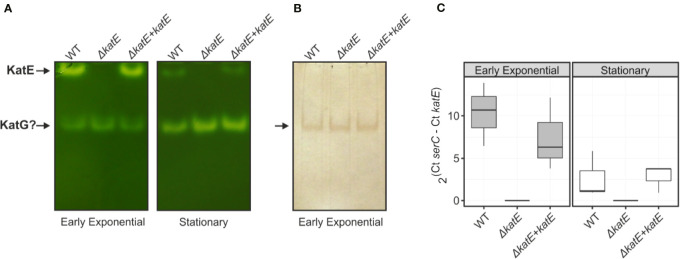
Catalase/peroxidase activity patterns and *katE* expression in *R. solanacearum*. Equal amounts of total soluble protein extracts (25 µg) from wild-type GMI1000 strain (WT), the *katE* deletion mutant (*ΔkatE*), and the complemented (*ΔkatE + katE*) strain grown in BG medium to early exponential or stationary growth phases were separated on non-denaturing polyacrylamide gels stained for catalase **(A)** and peroxidase **(B)** activities. **(C)** *katE* mRNA levels measured by quantitative real-time PCR from cultures grown in the same conditions described in A. In the Y-axis is represented the 2^ΔCt^ normalised expression from three biological replicas with two technical replicas each. Experiments in A and B were repeated at least three times with similar results.

To analyze the effect of *katE* deletion on *R. solanacearum* catalase activity, soluble protein extracts from cultures grown in BG medium to early exponential and stationary phase were separated on non-denaturing polyacrylamide gels and stained for catalase activity. As shown in **Figure 1A**, we detected two distinct catalase bands at both growth stages in the wild-type strain GMI1000. On the contrary, the upper, slow-migrating band was completely absent in the *ΔkatE* mutant, suggesting that this band corresponds to the KatE isozyme. As a final proof that the *RSp1581* open reading frame is functional and encodes this enzyme, complementation of *ΔkatE* with a single copy of the open reading frame under its own promoter restored the catalase activity pattern. Soluble protein extracts of the wild type, *ΔkatE* and complemented (*ΔkatE + katE*) strains were also run in parallel on a non-denaturing polyacrylamide gel stained for peroxidase activity (**Figure 1B**). This assay revealed that the fast-migrating catalase band detected in all three strains exhibits peroxidase activity as well, suggesting that it corresponds to one of the KatG isozymes identified in the *R. solanacearum* genome (Salanoubat et al., 2002). In adittion, the upper KatE band did not appear in the peroxidase assay further corroborating its monofunctional enzymatic nature.

The activity levels of KatE observed in native gels (**Figure 1A**) seemed to indicate that expression of this gene in *R. solanacearum* is regulated by growth phase, as previously reported for other bacterial species (Loewen, 1997; Vattanaviboon and Mongkolsuk, 2000; Tondo et al., 2010). To test this hypothesis, we measured the *katE* mRNA levels in early exponential and stationary phase cultures by quantitative real-time RT-PCR. As illustrated in **Figure 1C**, mRNA levels of *katE* significantly decreased in stationary wild type cells, being approximately 5-fold lower in the stationary phase with respect to early exponential growth phase. This expression pattern was similar in the complemented *ΔkatE* strain whereas expression was undetectable in the *katE* mutant.

### *KatE* Expression Is Transcriptionally Activated by the HrpG Regulator

**Figure 2:**
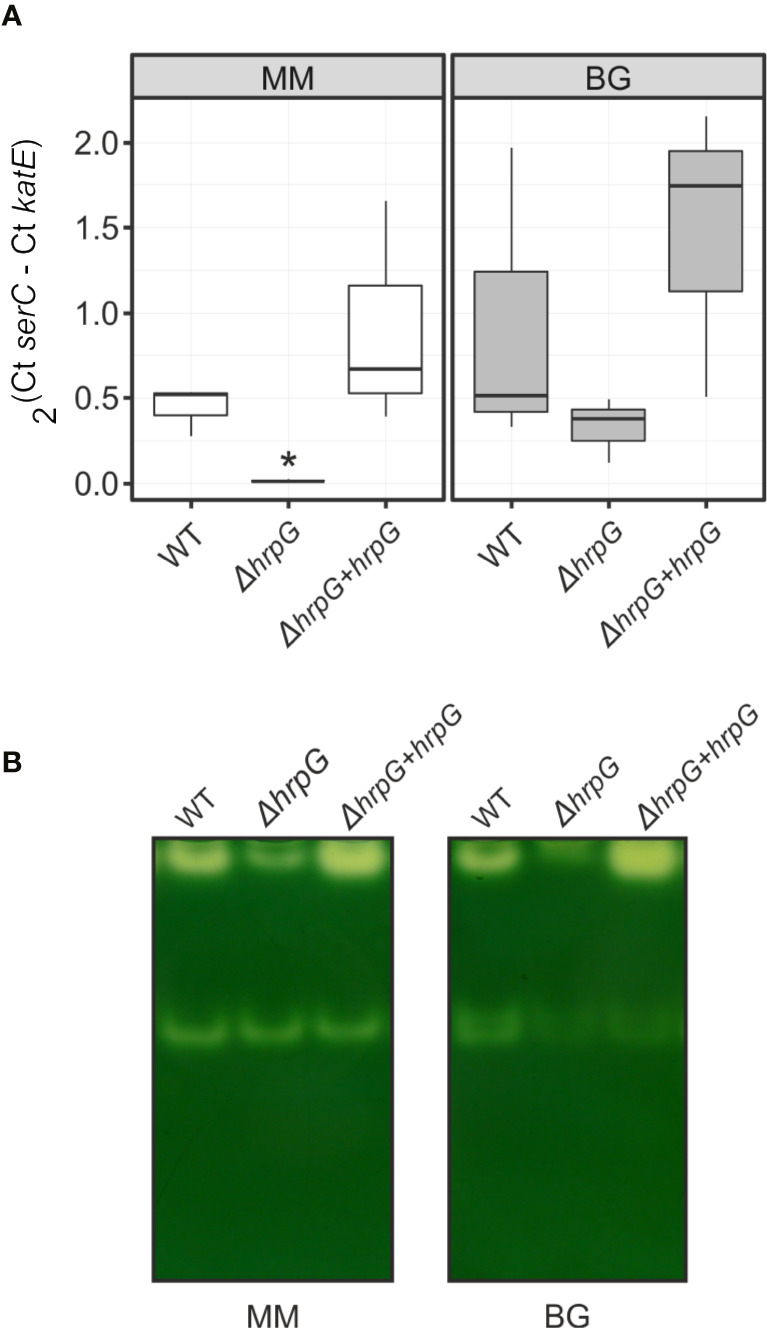
Expression of *katE* in the wild-type (WT), *ΔhrpG* mutant, and complemented overexpressing (*ΔhrpG + hrpG*) strains. **(A)** mRNA levels of *katE* were measured by quantitative real-time PCR in RNAs extracted from *R. solanacearum* cells grown in rich BG or minimal medium supplemented with glutamate. In the Y-axis is represented the 2^ΔCt^ normalised expression from three biological replicas with two technical replicas each. The asterisk indicates the strain showing statistically significant differences to the other two strains tested in the same condition (95% Tukey HSD test). **(B)** Catalase activity patterns in native gels. Equal amounts of soluble proteins (25 µg) were separated by 8% non-denaturing PAGE and stained for catalase activity. A simultaneously run Coomassie-stained gel (not shown) indicated equal protein loadings between samples. This experiment was repeated three times with similar results.

Using genome-wide expression analyses in *R. solanacearum*, we previously identified *katE* among a group of virulence and environmental adaptation genes specifically regulated by the HrpG transcriptional regulator (Valls et al., 2006). To better investigate the role of HrpG in the regulation of *katE*, we measured *katE* transcript levels in the wild-type GMI1000 strain, a *hrpG* deletion mutant (*ΔhrpG*) and the complemented mutant strain overexpressing this regulator (*ΔhrpG + hrpG*). *katE* mRNA levels were significantly lower in the *ΔhrpG* strain with respect to the wild-type or the complemented overexpressing strain (**Figure 2A**). This effect was more pronounced (significant differences in 95% Tukey HSD test) in minimal medium -known to specifically induce HrpG activity- than in cells grown in rich BG medium (**Figure 2A**). To evaluate the influence of this regulation at the protein level, we then measured the effect of *hrpG* on the catalase activity. Measurements of catalase activity in native polyacrylamide gels revealed the same expression pattern obtained for *katE* transcripts, with markedly lower levels in the *ΔhrpG* background that could be complemented by overexpression of this regulator (**Figure 2B**). These results show a clear correlation between *hrpG* and *katE* transcript levels and with the catalase activity as well.

### *R. solanacearum* KatE Activity Is Enhanced Upon H_2_O_2_ Treatment and Protects Against Oxidative Stress

**Figure 3:**
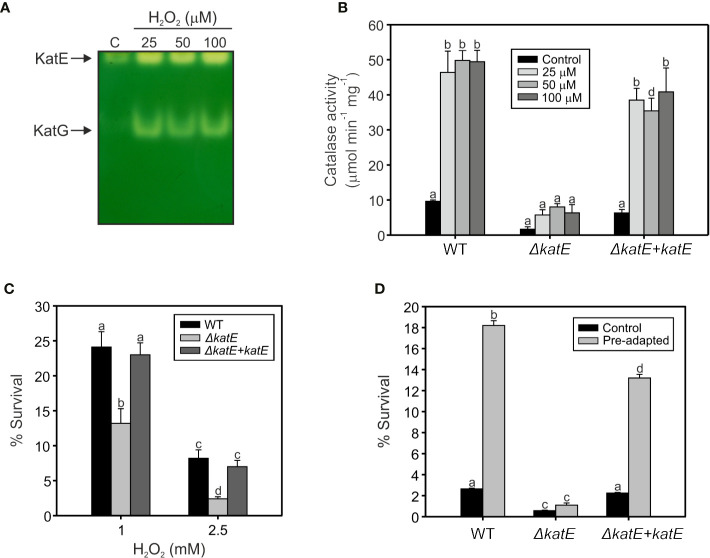
Hydrogen peroxide response in wild type, the *ΔkatE* mutant, and its complemented counterpart. **(A)** Catalase activities detected after exposure to sub-lethal levels of hydrogen peroxide. Equal amounts (15 µg) of soluble protein extracts from early exponential phase cultures exposed to 25, 50, and 100 µM of H_2_O_2_ for 60 min, and from an untreated control (C), were separated on a native polyacrylamide gel stained for catalase activity. **(B)** Catalase activities quantified through H_2_O_2_ decomposition in soluble cell extracts obtained from cells treated as in A. **(C)** Sensitivity of *R. solanacearum* strains to 1 and 2.5 mM H_2_O_2_. Cells in early exponential phase of growth were exposed to the indicated concentrations of H_2_O_2_ for 15 min. The number of Colony Forming Units (CFU) was determined for each culture before and after the peroxide treatment by plating of appropriate dilutions. The percentage of survival is defined as the number of CFU after treatment divided by the number of CFU prior to H_2_O_2_ exposure ×100. **(D)** Sensitivity of pre-adapted cultures of WT, *ΔkatE*, and the complemented *ΔkatE + katE* strains to 5 mM H_2_O_2_. Exponential phase cultures were first adapted with 100 µM H_2_O_2_ for 60 min and then exposed to 5 mM H_2_O_2_ for 15 min. The number of CFU was determined for each culture before and after the 5 mM H_2_O_2_ treatment by plating of appropriate dilutions and percentage of survival calculated as the number of CFU after treatment divided by the number of CFU prior to treatment ×100. Data represent the mean and standard deviation of three independent experiments. Different letters indicate significant differences among strains and/or treatment according to the Tukey’s multiple comparison test (p<0.0001).

To assess the involvement of catalases in the *R. solanacearum* oxidative stress response, we exposed early exponential phase cultures to a range of sub-lethal doses (25, 50, and 100 µM) of H_2_O_2_ for 1 h and the catalase activity patterns were compared to untreated control cultures on native polyacrylamide gels. As shown in **Figure 3A**, the activity of the two detected catalase bands increased upon H_2_O_2_ treatment, suggesting a clear induction of both isoforms under oxidative stress. To further evaluate the contribution of KatE to this response, we quantified the catalase activity levels in the wild type, the *ΔkatE* mutant and the complemented (*ΔkatE + katE*) strains grown in the conditions previously stated (**Figure 3B**). H_2_O_2_ treatment caused a ~5-fold increase of catalase activity in the wild-type strain, being this increment almost equivalent at all H_2_O_2_ concentrations tested. On the contrary, no catalase induction was observed in the *katE* mutant after peroxide exposure, suggesting an impaired ability to face the oxidative challenge.

Resistance of bacterial cells to lethal doses of H_2_O_2_ was then evaluated. As illustrated in **Figure 3C**, the *ΔkatE* mutant exhibited increased sensitivity to the oxidant compared to the parental wild-type strain, a phenotype that was more pronounced at higher H_2_O_2_ doses and maximal at the highest concentration tested (2.5 mM). Moreover, pre-adaptation of the cultures with a sub-lethal concentration of H_2_O_2_ (100 µM) led to a significant increase in the resistance of wild-type cells to an elevated dose (5 mM) of the agent (**Figure 3D**). This effect, commonly known as *adaptive response*, was not observed in the *ΔkatE* strain, which did not evidence higher tolerance to the oxidant after the adaptation treatment, reinforcing the notion that *katE* encodes the only catalase activity that contributes to bacterial adaptation to an oxidative environment.

### Biofilm Formation Is Affected by the Deletion of *katE*

**Figure 4:**
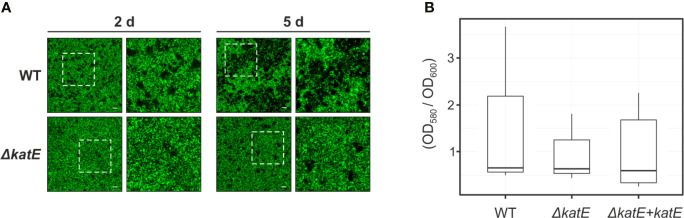
Effect of *katE* disruption on *R. solanacearum* biofilm formation ability. **(A)** GFP-labeled wild-type and *ΔkatE* strains were grown on chambered cover slides and visualized under confocal laser scanning microscopy after 2 and 5 days of bacterial growth. Left panels show the biofilms developed at the bottom of the chambered cover slides with a magnification of 400X and right panels show a 2X zoom of the regions marked in the previous panels. Scale bars, 50 μm. **(B)** Biofilm quantification. Bacterial suspensions were grown for 24 h in 96 well plates at 30°C, stained with crystal violet and the biofilm was quantified as the OD_580_ normalized by the bacterial growth measured at OD_600_. Boxplots of the values obtained per each tested strain from 5-6 biological replicas (N=5-6) are presented.

Bacterial antioxidant activities have been shown to influence biofilm formation (Kim et al., 2006; Simmons and Dybvig, 2015; Tondo et al., 2016). To analyze the structural characteristics of the *R. solanacearum* biofilm, we generated Green Fluorescent Protein (GFP)-labeled strain derivatives (**Table 1**) and observed their growth development on chambered cover glass slides by confocal laser scanning microscopy over a 5-day period. At two days post inoculation (dpi), formation of cell aggregates was apparent for the wild-type strain (**Figure 4A**), and a well-established biofilm with more complex structures was clearly observed at 5 dpi. In contrast, the *katE* mutant failed to form a structured biofilm after 5 days, exhibiting minor aggregation and reduced interstitial spaces. Besides observing the biofilm structure, we also quantified the amount of biofilm produced by measuring the intensity of crystal violet staining after growth on 96-well plates. As shown in **Figure 4B**, these experiments resulted in comparable quantities of biofilm in the wild type, the *katE* mutant and its genetically complemented derivative, demonstrating that KatE influences the development of biofilm structures but does not alter the overall amount of biofilm produced.

### Pathogenicity Tests

**Figure 5:**
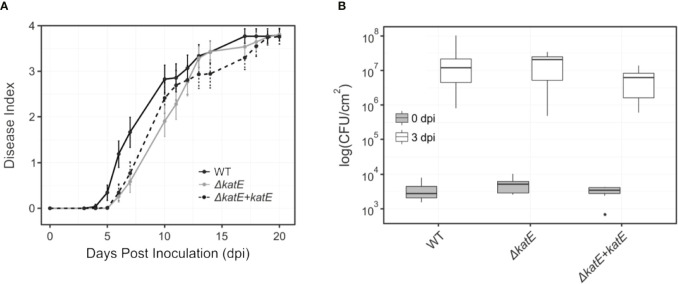
Effect of *katE* in *R. solanacearum* virulence and fitness in the apoplast. **(A)** Bacterial pathogenicity assay on tomato. Wilting symptoms were recorded after soil-drench inoculation with the wild type *R. solanacearum* GMI1000 strain (black line), the *katE* disruption mutant derivative (grey line) and its complemented strain (dotted black line). Disease symptoms are plotted over time in a scale ranging from 0 (no symptoms) to 4 (wilted plant). Each data point represents the average of 20-25 plants and their standard errors. Three independent biological replicates were performed with similar results. **(B)** Bacterial growth in leave tissues. Tomato plants were vacuum infiltrated with 10^5^ CFU/mL suspensions of GMI1000, the *katE* mutant, and the complemented strain. Leaf disks were sampled at day 0 and 3 post inoculation, and bacterial counts in the tissue were determined as CFU from plated dilutions normalized to the disk area sampled (N=3). All experiments were repeated three times with similar results.

As mentioned previously, *katE* transcription is activated by the master regulator of pathogenicity HrpG (**Figure 2**). In addition, our preliminary data show that *katE* from *R. solanacearum* strain UY031 is highly expressed when the bacterium grows in the plant apoplast and in the xylem (unpublished data). This information, together with our finding that catalase activity was key to survive oxidative stress led us to test whether it is required for *R. solanacearum* GMI1000 pathogenicity on tomato, its natural host. Plants of the susceptible tomato cultivar Marmande were inoculated with suspensions of the wild type, mutant, and complemented strains by soil drenching and symptom appearance was recorded over time (**Figure 5A**). No statistical differences in wilting symptoms in plants inoculated with the wild type, the *katE* disruption mutant or the complemented strain were observed in three biological replicas, suggesting no major role of the gene in the virulence of *R. solanacearum* GMI1000. The importance of apoplastic ROS led us to quantify whether bacterial fitness was affected during growth in this plant compartment. To this end, we infiltrated susceptible tomato leaves with solutions of the wild type, the *ΔkatE*, and the complemented strain and quantified bacterial concentrations in recovered leaf disk samples immediately after inoculation and at three days post inoculation (dpi). Results from a representative experiment are presented in **Figure 5B** and show that no differences in bacterial multiplication in the apoplast were observed for any of the three tested strains.

## Discussion

It has been shown that hydrogen peroxide is a central component of the oxidative burst during plant-pathogen interaction, as it accumulates in plants attacked by pathogenic microorganisms including fungi, bacteria and viruses (Baker and Orlandi, 1995; Wojtaszek, 1997). In this context, the antioxidant system adequacy by the invading microorganism must be fundamental to minimize the oxidative stress generated by the host plant, thus achieving the establishment of the infection. In this work, we demonstrated that monofunctional KatE and bifunctional KatG catalase activities can be detected in *R. solanacearum* soluble protein extracts using non-denaturing polyacrylamide gels (**Figure 1A**). Furthermore, a single mutant in the *katE* gene was generated and genetically complemented corroborating that the upper band revealed in the native gel corresponds to the KatE catalase.

We evaluated catalase activities during the different growth phases, detecting that the monofunctional catalase was induced during exponential growth (**Figures 1A, C**). These results collectively suggest that *katE* expression is growth phase regulated at the transcriptional level. Similar results were previously reported for other bacteria such as *E. coli, Xanthomonas campestris* pv. *campestris* and *Xanthomonas citri* subsp. *citri*, although the expression pattern of particular catalase isozymes may vary between species (Loewen, 1997; Vattanaviboon and Mongkolsuk, 2000; Tondo et al., 2010). In *X. citri* subsp. *citri*, *katE* gene was also regulated by growth phase but contrary to the pattern observed in *R. solanacearum*, it exhibited an strong induction in stationary phase cells (Tondo et al., 2010).

Our results show that *katE* is transcriptionally activated by HrpG but also responds to other inducing cues besides the growth phase, as shown by the higher transcriptional output observed upon growth in BG rich medium than in minimal medium, a condition known to induce HrpG activity (**Figure 2A**). This specific induction in rich medium independently of HrpG is corroborated by the high *katE* mRNA levels in the *ΔhrpG* mutant strain grown in this medium. Finally, *katE* expression seems to be controlled mostly at the transcriptional level, as the levels of the KatE enzyme mostly correlate with its mRNA abundance, although protein stability may be increased post-translationally in minimal medium, as indicated by the fact that it can be detected in the *ΔhrpG* mutant strain grown in this condition, where it shows minimal transcription levels (**Figures 2A, B**).

On the other hand, we studied the participation of the two *Ralstonia* catalases in the resistance against the oxidizing compound hydrogen peroxide. *R. solanacearum* exponential cultures were exposed to sub-lethal doses of peroxide, detecting a clear induction of both catalase isoforms under oxidative stress (**Figure 3A**). These results are in agreement with those obtained by Florez-Cruz and Allen, who observed an OxyR-dependent induction of *katE* and *katG* mRNA levels after exposure to H_2_O_2_ (Flores-Cruz and Allen, 2009). Here, the contribution of KatE to this response was analyzed (**Figure 3B**). Quantification of *R. solanacearum* catalase activity in the *ΔkatE* mutant showed that it is almost residual and that its induction is undetectable (**Figure 3B**). The catalase activity was recovered in the complemented strain, where *katE* was reintroduced into the mutant background, and showing that KatE plays a significant role in the *R. solanacearum* protection to oxidative stress. To prove this, bacterial cultures were confronted to lethal doses of H_2_O_2_ detecting that the *ΔkatE* mutant was more susceptible to the oxidative compound than the wild type strain (**Figure 3C**). This is in agreement with the reported observations that disruption of the monofunctional catalase *katE* in *X. citri* subsp. *citri* and *katB* in *Pseudomonas syringae* pv. tomato DC3000 rendered these bacteria more susceptible to oxidative stress (Tondo et al., 2010; Guo et al., 2012). The other monofunctional catalase in *P. syringae* pv. tomato (KatE), which is clearly less induced by exposure to exogenous H_2_O_2_, also showed a minor role in resistance to the oxidative compound (Guo et al., 2012).

The adaptive response to oxidative agents has been previously proposed to play a fundamental role in plant-pathogen interactions, allowing bacteria to withstand increased oxidative stress conditions (Ausubel, 2005). Exposure to sub-lethal concentrations of oxidative stress agents usually have a priming effect on bacteria, which then tolerate higher doses of the same oxidant (adaptive response), and even others (cross-protection). These responses are due to the induction of numerous genes involved in oxidant removal and damage repair, including catalases (Demple, 1991; Tartaglia et al., 1991). Evaluation of this response in *R. solanacearum* showed that *katE* mutant does not significantly induce catalase activity upon treatment with low doses of H_2_O_2_ and its remained activity is not enough to protect bacteria against higher doses of the oxidant (**Figures 3B, D**). Consequently, even though KatG activity was found induced in peroxide-treated cultures according to in-gel catalase staining, our results suggest a minor role for the additional KatG catalases in the response to H_2_O_2_, being KatE the only catalase activity contributing to the bacterial adaptive response to an oxidative environment.

Our finding that KatE catalase activity was essential for survival in oxidative environments and the fact that ROS is a major player in plant defence responses (Wojtaszec, 1997; Flores-Cruz and Allen, 2009) led us to investigate its role in bacterial virulence. Surprisingly, we found no effect of the *katE* mutation on pathogenicity assays on tomato (**Figure 5A**). This could be due to the limited sensitivity of soil drench inoculation and disease scoring to detect minor differences in bacterial pathogenicity. An alternative explanation is that ROS accumulate mainly in the apoplast (Lamb and Dixon, 1997) and *R. solanacearum* grows mostly inside the xylem vessels of host plants. Thus, we measured the capacity of the bacterium to multiply in the tomato apoplast as a more quantitative measurement of its virulence and fitness. Again, disruption of *katE* did not cause any effect (**Figure 5B**). Although bacterial multiplication in the host is not always correlated with its aggressiveness (Angot et al., 2006), this result was somehow unexpected due to the important role played by the KatE catalase in *in vitro* protection to oxidative stress, a condition that is commonly encountered by bacteria inside the plant host (Lamb and Dixon, 1997).

In addition, the *katE* mutant strain did not show reduced ability to produce biofilms, another important trait for the wilting disease development (**Figure 4**). Biofilm-growing cells usually experience endogenous oxidative stress and many antioxidant systems were shown to be induced under this growth condition (Resch et al., 2005; Ram et al., 2005; Mikkelsen et al., 2007; Shanks et al., 2007; Chung et al., 2016). In fact, the role of catalase and superoxide dismutase in the development of mature biofilms was previously demonstrated in *X. citri* subsp. *citri* and *E. coli*, respectively (Kim et al., 2006; Tondo et al., 2016). According to our results, disruption of *katE* in *R. solanacearum* only alters the structure of the biofilm produced on an abiotic surface, but not the overall quantity of biofilm production. This is in agreement with previous reports indicating that perturbations of the physiological steady-state levels of ROS or the addition of catalase to the medium affects the quality and structural characteristics of the biofilms developed by *Azotobacter vinelandii* (Villa et al., 2012) and *Mycoplasma pneumoniae* (Simmons and Dybvig, 2015), with diverse effects on the amounts of biofilm produced.

However, the minor role that KatE seems to play *in planta* is in agreement with a previous screening for *R. solanacearum* genes essential for growth *in planta*, in which *katE* was not identified (Brown and Allen, 2004). The two possible explanations for the undetectable effect of *R. solanacearum katE* disruption on plant infection are, that ROS are not key players in the defence against this pathogen in tomato cv Marmande or that functional redundancy with other genes with catalase activity exists. The three catalases in *P. syringae* pv. tomato DC3000 are all plant induced and play non-redundant roles in virulence (Guo et al., 2012). Our results corroborate the hypothesis proposed by Guo *et al*. that catalases play different roles in each plant pathogen where they independently adapted to overcome the plant defensive production of H_2_O_2_. Our ongoing characterisation of the KatG catalases-peroxidases will be essential to shed light into this question.

## Data Availability Statement

All datasets presented in this study are included in the article/**Supplementary Material**.

## Author Contributions

MLT, MV, and EO conceived and designed the work. MLT, RP-J, and AV performed the experiments. LP and RP-J contributed to statistical analyses. EO and MV provided reagents and materials. All authors contributed to analysis and interpretation of results. MLT, RP-J, MV, and EO wrote the manuscript. All authors contributed to the article and approved the submitted version.

## Funding

This work was supported by the Agencia Nacional de Promoción Científica y Tecnológica (ANPCyT PICT 2014-2487 to EO and PICT 2014-2485 to MLT) and the Spanish Ministry of Economy and Competitiveness (AGL2016-78002-R and PID2019-108595RB-I00 to MV). We also acknowledge financial support from the “Severo Ochoa Program for Centers of Excellence in R&D” (SEV/2015/0533), and the CERCA Program from the Catalan Government (Generalitat de Catalunya). The funders had no role in study design, data collection and analysis, decision to publish, or preparation of the manuscript. EO and MLT are staff members and AV is Fellow of the Consejo Nacional de Investigaciones Científicas y Técnicas (CONICET, Argentina).

## Conflict of Interest

The authors declare that the research was conducted in the absence of any commercial or financial relationships that could be construed as a potential conflict of interest.

## Supplementary Material

Supplementary Figure 1 Growth curves of *R. solanacearum* GMI1000 wild-type (WT), *katE* mutant (*ΔkatE*) and complemented (*ΔkatE + katE*) strains in BG medium. *R. solanacearum* cultures were grown aerobically at 28°C with shaking at 200 rpm. Aliquots were taken at the indicated times and measured for colony-forming capacity by serial dilution and plating on BG-agar. Colonies were counted after 48 h incubation at 28°C.
