# Supplementary figures and images for: KatE From the Bacterial Plant Pathogen Ralstonia solanacearum Is a Monofunctional Catalase Controlled by HrpG That Plays a Major Role in Bacterial Survival to Hydrogen Peroxide
Source: Front Plant Sci. 2020 Jul 31;11:1156. doi: 10.3389/fpls.2020.01156 (PMC7412880; doi:10.3389/fpls.2020.01156)

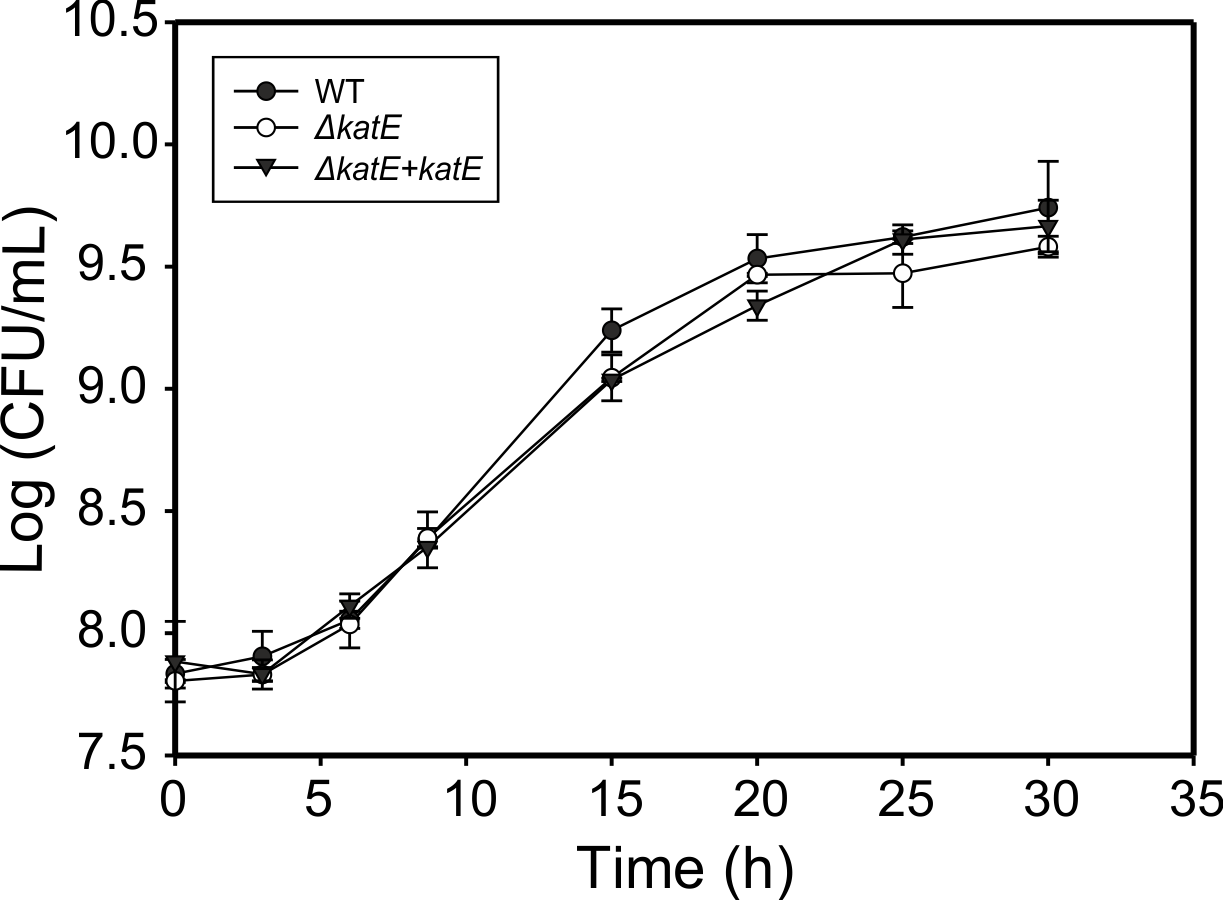

Supplement: Supplementary Figure 1 — Growth curves of R. solanacearum GMI1000 wild-type (WT), katE mutant (ΔkatE) and complemented (ΔkatE + katE) strains in BG medium. R. solanacearum cultures were grown aerobically at 28°C with shaking at 200 rpm. Aliquots were taken at the indicated times and measured for colony-forming capacity by serial dilution and plating on BG-agar. Colonies were counted after 48 h incubation at 28°C. [file Image_1.tif]
